# Prevalence of metabolic syndrome in Bangladesh: a systematic review and meta-analysis of the studies

**DOI:** 10.1186/s12889-018-5209-z

**Published:** 2018-03-02

**Authors:** Mohammad Ziaul Islam Chowdhury, Ataul Mustufa Anik, Zaki Farhana, Piali Dey Bristi, B. M. Abu Al Mamun, Mohammad Jasim Uddin, Jain Fatema, Tanjila Akter, Tania Akhter Tani, Meshbahur Rahman, Tanvir C. Turin

**Affiliations:** 1 Department of Community Health Sciences, University of Calgary, TRW Building (3rd Floor), 3280 Hospital Drive NW, Calgary, AB T2N 4Z6 Canada; 2 Department of Statistics, Shahjalal University of Science and Technology, Sylhet, Bangladesh; 3 Department of General Surgery, North East Medical College, Sylhet, Bangladesh; 4 Department of Clinical Pathology, Sylhet MAG Osmani Medical College Hospital, Sylhet, Bangladesh; 5 Department of Family Medicine, University of Calgary, Calgary, AB Canada

**Keywords:** Metabolic syndrome, Prevalence, Bangladesh

## Abstract

**Background:**

Metabolic syndrome (MS) is a cluster of health problems that set the stage for serious health conditions and places individuals at higher risk of cardiovascular disease, diabetes and stroke. The worldwide prevalence of MS in the adult population is on the rise and Bangladesh is no exception. According to some epidemiological study, MS is highly prevalent in Bangladesh and has increased dramatically in last few decades. To provide a clear picture of the current situation, we conducted a systematic review and meta-analysis with an objective to assess the prevalence of metabolic syndrome among the Bangladeshi population using data already published in the scientific literature.

**Methods:**

We searched MEDLINE, EMBASE and PubMed and manually checked references of all identified relevant publications that described the prevalence of MS in Bangladesh. Random effects meta-analysis was used to pool the prevalence. Heterogeneity was explored using formal tests and subgroup analyses. Study quality and publication bias was also explored.

**Results:**

Electronic and grey literature search retrieved 491 potentially relevant papers. After removing duplicates, reviewing titles and abstracts and screening full texts, 10 studies were finally selected. Most of the studies were conducted in rural populations and study participants were mostly females. The weighted pooled prevalence of metabolic syndrome regardless of gender and criteria used to define metabolic syndrome, was 30.0% with high heterogeneity observed. Weighted pooled prevalence of metabolic syndrome is higher in females (32%) compared to males (25%) though not statistically significant (*p* = 0.434). Prevalence was highest (37%) when Modified NCEP ATP III criteria was used to define MS, while it was lowest (20%) when WHO criteria was used. In most cases, geographical area (urban/rural) was identified as a source of heterogeneity between the studies. Most of the studies met study quality assessment criteria’s except adequate sample size criteria and evidence of small study effect was also detected.

**Conclusions:**

The prevalence of metabolic syndrome is high and rising in Bangladesh. Strategies aimed at primary prevention are required to mitigate a further increase in the prevalence and for the reduction of the morbidity and mortality associated with metabolic syndrome.

**Electronic supplementary material:**

The online version of this article (10.1186/s12889-018-5209-z) contains supplementary material, which is available to authorized users.

## Background

Metabolic syndrome (MS) is a cluster of health problems that include abdominal fat, high blood pressure, high triglycerides, elevated blood sugar and low HDL cholesterol. The underlying causes of metabolic syndrome include overweight and obesity, insulin resistance, an unhealthy dietary pattern, physical inactivity, genetic factors and aging [1]. The worldwide prevalence of metabolic syndrome in the adult population is on the rise with an estimated prevalence of 20–25% [1]. Adults with metabolic syndrome are twice as likely to die from and are three times as likely to have a heart attack or stroke compared with people without the metabolic syndrome [1–5]. In addition, they have a five-fold greater risk of developing type 2 diabetes [6]. In this backdrop, metabolic syndrome is being considered as public health issue globally [7, 8].

Prevalence of non-communicable chronic diseases and associated mortality has also increased dramatically in Bangladesh in the last few decades [9, 10]. Bangladesh, a developing country with fast economic growth, has been experiencing rapid urbanization for the past several decades [9, 11]. This development and urbanization raises the concern that the chronic disease burden may show an increasing trend in future, especially due to altering food habit including increased access to and popularity of processed food, irregular meal times, less physical activity, etc. [12].

Though a handful of studies were conducted about metabolic syndrome in Bangladeshi population [13, 14], few studies have described the prevalence of metabolic syndrome and its related factors, hence restricting the quality of information available on the magnitude of this problem in Bangladesh. In Bangladesh, there is no population-based surveillance system to track non-communicable chronic disease. Also there is a lack of national population based surveys or central administrative health data’s to obtain accurate information’s on prevalence of disease like metabolic syndrome. Having comprehensive information about the prevalence of metabolic syndrome can be very effective for planning and executing preventive strategies for such diseases. To provide a clear picture of the current situation, we conducted a systematic review and meta-analysis with an objective to assess the prevalence of metabolic syndrome among the Bangladeshi population using data already published in the scientific literature. The performance of meta-analysis will help to combine the existing data on the prevalence of metabolic syndrome and explore possible heterogeneity between studies.

## Methods

### Data sources and searches

We systematically searched MEDLINE, EMBASE and PubMed (from inception to February 10, 2017) for studies on prevalence of metabolic syndrome among Bangladeshi population. We also searched the reference lists of all identified relevant publications for information about other potential studies. We limited inclusion to studies published in English. The search strategy focused on three key elements: metabolic syndrome, prevalence and Bangladesh. The search strategy is provided in detail in Table 1.

**Table 1:** Search strategy used in different databases

MEDLINE	PubMed	EMBASE
1. exp Metabolic Syndrome X/ 2. exp. Syndrome/ 3. Insulin resistance syndrome.mp. 4. exp. Hypertension/ 5. high blood pressure.mp. 6. exp. Hyperlipidemias/ 7. lipid disorder.mp. 8. 4 or 5 9. 6 or 7 10. exp. Hyperglycemia/ 11. exp. Diabetes Mellitus/ 12. high blood sugar.mp. 13. 10 or 11 or 12 14. exp. Obesity, Abdominal/ 15. 8 and 9 16. 8 and 14 17. 8 and 13 18. 9 and 14 19. 9 and 13 20. 13 and 14 21. exp. Prevalence/ 22. exp. Bangladesh/ 23. 1 or 2 or 3 or 15 or 16 or 17 or 18 or 19 or 20 24. 21 and 22 and 23	Search (((((((((((Metabolic Syndrome) OR Syndrome) OR Insulin resistance syndrome) OR ((((Hypertension) OR high blood pressure)) AND ((Hyperlipidemia) OR lipid disorder))) OR ((((Hypertension) OR high blood pressure)) AND (((hyperglycemia) OR diabetes mellitus) OR high blood sugar))) OR ((abdominal obesity) AND ((Hypertension) OR high blood pressure))) OR ((((Hyperlipidemia) OR lipid disorder)) AND (((hyperglycemia) OR diabetes mellitus) OR high blood sugar))) OR ((abdominal obesity) AND ((Hyperlipidemia) OR lipid disorder))) OR ((abdominal obesity) AND (((hyperglycemia) OR diabetes mellitus) OR high blood sugar)))) AND prevalence) AND Bangladesh	1. exp Metabolic Syndrome X/ 2. exp. Syndrome/ 3. Insulin resistance syndrome.mp. 4. exp. Hypertension/ 5. high blood pressure.mp. 6. exp. Hyperlipidemia/ 7. lipid disorder.mp. 8. exp. hyperglycemia/ 9. exp. diabetes mellitus/ 10. high blood sugar.mp. 11. exp. abdominal obesity/ 12. 4 or 5 13. 6 or 7 14. 8 or 9 or 10 15. 12 and 13 16. 12 and 14 17. 11 and 12 18. 13 and 14 19. 11 and 13 20. 11 and 14 21. 1 or 2 or 3 or 15 or 16 or 17 or 18 or 19 or 20 22. exp. prevalence/ 23. exp. Bangladesh/ 24. 21 and 22 and 23

### Study selection

Two reviewers independently identified potentially eligible articles by performing an initial screen of titles and abstracts. Articles were considered for inclusion if they reported data from an original study (review articles were excluded) and reported on the prevalence of metabolic syndrome in Bangladesh. We used broad inclusion criteria to provide a comprehensive systematic review of the topic. There were no restrictions on study type (e.g., cohort study, cross sectional study), geographic region (e.g., urban, rural) or age ranges. Studies that reported the prevalence of metabolic syndrome in the general population were included. We excluded those studies where prevalence of metabolic syndrome was measured only in specific clinical population (e.g. individuals with hypertension or diabetes) regardless of how they define those clinical population. However, we did not exclude studies where hypertension or diabetes in the general population was used as a criteria to define metabolic syndrome in order to measure its prevalence. We excluded the animal and biomedical studies that did not report prevalence of metabolic syndrome, non-human studies, and studies that only focused on the prevalence of a component of metabolic syndrome (e.g. prevalence of hypertension). Articles were retained when anyone of the reviewers believed that it should be retained or when there was uncertainty as to eligibility on the basis of title and abstract alone. Agreement between reviewers was quantified. Any disagreement between reviewers was resolved by consensus. Selected articles were subsequently screened on the basis of a full-text review. We considered any definition of metabolic syndrome.

### Data extraction

From the finally selected articles, the following information’s were extracted: author and year of publication, age range of the participants, gender and number of participants, area (urban/rural) in which the study was carried out, sample selection procedure, study design, criteria for diagnosis of metabolic syndrome, and the prevalence of metabolic syndrome and its components. Two reviewers independently extracted data using a standardized form. Study quality was assessed by each reviewer, according to the Joanna Briggs Institute guidance on conducting prevalence and incidence reviews [15, 16] and following data’s were extracted from each study for quality assessment purpose: was the sample representative of the target population, were study participants recruited in an appropriate way, was the sample size adequate, were the study subjects & setting described in detail, was the data analysis conducted with sufficient coverage of the identified sample, were objective, standard criteria used for measurement of the condition, was the condition measured reliably, and are all the important confounding factors/subgroups/differences identified and accounted for.

### Data analysis

We grouped studies on the basis of the criteria used to diagnosis of metabolic syndrome, whether National Cholesterol Education Program Adult Treatment Panel III criteria (NCEP ATP III), modified NCEP ATP III criteria, International Diabetes Federation criteria (IDF) or World Health Organization criteria (WHO). We used random effects meta-analysis to obtain the weighted average prevalence with 95% CIs for studies. While defining metabolic syndrome, studies were not all consistent with the way they define different individual components of metabolic syndrome. For example, some studies used the term elevated blood pressure while other used the term hypertension. The threshold for elevated blood pressure or hypertension was also not consistent. Same applies in the case of elevated fasting blood glucose or diabetes also. Similarly, the terms elevated waist circumference, central obesity or obesity waist was used interchangeably with no unique cut-off to measure abdominal obesity. Even in one study, BMI was substituted for waist circumference. Despite some minor differences exists in defining and using thresholds in different components of metabolic syndrome, we ignored such differences while conducting meta-analysis on the prevalence of metabolic syndrome assuming that such minor difference won’t impact the overall result. We also grouped studies on the basis of the gender and age of the study participants and assessed temporal change on metabolic syndrome prevalence.

Heterogeneity was assessed using the Cochran Q and the I^2^ statistic and was explored using meta-regression and stratified analyses according to the gender of the study participants and criteria used to diagnosis metabolic syndrome. Small study effects were examined using funnel plot and Egger’s test. Inter rater reliability was measured. All statistical analyses were performed using Stata version 13.1 (Stata Corp, College Station, TX) using the metaprop, metareg, metabias, and metafunnel commands.

## Results

**Fig. 1:**
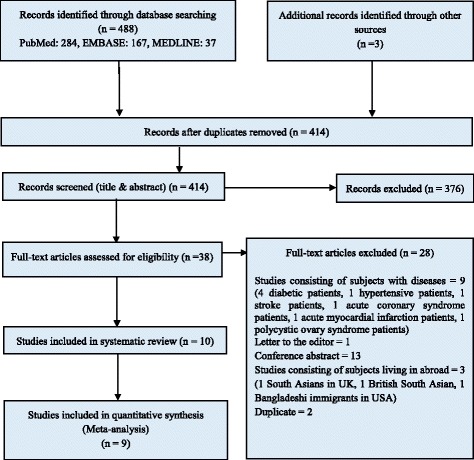
PRISMA diagram for systematic review of studies that evaluated the prevalence of metabolic syndrome (MS) in the Bangladeshi population

Our electronic search retrieved 488 and grey literature search retrieved 3 potentially relevant papers on the prevalence of metabolic syndrome in Bangladesh. After removing duplicates, reviewing titles and abstracts, 38 articles remained for full text screening; main reason for exclusion was irrelevance with our study objective. Of the 38 articles screened (titles and abstracts), 28 were excluded for the following reasons: 9 were conducted on subjects with diseases, 14 was not an original article (13 conference abstract and 1 letter), 3 carried out on Bangladeshi population living abroad and 2 duplicate studies. Therefore, 10 studies were finally selected for the present systematic review. There was good agreement (89.47%) between reviewers on the final articles eligible for inclusion. The selection processes for the articles are shown in Fig. 1.

A summary describing the characteristic of the selected studies on the prevalence of metabolic syndrome in Bangladesh is presented in Table 2. Most of the studies were conducted in rural populations (8 out of 10). Only two studies [13, 17] conducted in urban population. Almost all the studies were local community based studies. Five of the studies were conducted in northern Rangpur region, four studies conducted in central Dhaka region and one study conducted in south-eastern Chittagong region. No studies were conducted in Sylhet, Khulna, Barisal and Rajshahi region (Additional file 1: Figure S1). Various criteria had been used to diagnose metabolic syndrome. Four studies used the criteria for diagnosing metabolic syndrome proposed by the National Cholesterol Education Program Adult Treatment Panel III (NCEP-ATP III) [18–21]; one the criteria of the International Diabetes Federation (IDF) [17]; one the criteria of the modified NCEP-ATP III [22]; one study used both modified NCEP-ATP III and IDF criteria [23]; two study used Modified NCEP-ATP III, IDF and WHO criteria [13, 14]; and one study used NCEP ATP III, Modified NCEP ATP III and IDF criteria [24]. Five studies carried out only on female participants while five studies on both male and female participants. Study design was cross-sectional in all the studies and sample was selected using stratified multi-stage random sampling procedure by 6 studies and using simple random sampling procedure by 4 studies.

**Table 2:** Characteristics of studies that evaluated the prevalence of metabolic syndrome (MS) in the Bangladeshi population

Study	Year Published	Age Range	Gender	Sample Size and Type	Study Area (Urban/Rural)	Sampling Method	Study Design	Criteria for Diagnosis of MS	Overall Prevalence of MS (%)	Prevalence of individual components of MS (%)
Akter et al. [18]	2012	15–75 years	Female	1423; Local Community-Based	Rural	Stratified multi-stage random sampling.	Community-based cross-sectional study	NCEP-ATP III	26.14 (age < 12 years at menarche), 25.73 (age 12–13 years at menarche), 22.13 (age > 13 years at menarche)	NR
Akter et al. [19]	2013	15–75 years	Female	1219; Local Community-Based	Rural	Stratified multi-stage random sampling.	Community-based cross-sectional study	NCEP-ATP III	NR	NR
Bhowmik et al. [23]	2015	≥ 20 years	Both	2293; Local Community-Based	Rural	Random sampling	Population based cross-sectional study	Modified NCEP-ATP III and IDF	30.7 (male 30.9, female 30.5) (Modified NCEP ATP III); 24.5 (male 19.2, female 27.5) (IDF)	Elevated waist circumference: 39.8; dyslipidemia: 28.7; hypertension: 15.5; mean fasting plasma glucose: 5.2
Rahim et at. [14]	2007	≥ 20 years	Both	3981; Local Community-Based	Rural	Simple Random Sampling	Cross-sectional survey	Modified NCEP-ATP III, IDF and WHO	20.7 (male 14.3, female 25.1) (Modified NCEP-ATP III); 11.2 (male 4.3, female 15.7) (IDF); 8.6 (male 7.6, female 9.2) (WHO)	NR
Jesmin et al. [20]	2013	≥ 15 years	Female	1802; Local Community-Based	Rural	Stratified multi-stage random sampling	Community based cross-sectional survey	NCEP-ATP III.	25.6	Elevated waist circumference: 8.46; High blood pressures: 28.16; Elevated fasting blood glucose: 35.39; Low HDL cholesterol: 84.14; High triglyceride: 29.49
Jesmin et al. [24]	2012	≥ 15 years	Female	1535; Local Community-Based	Rural	Stratified multi-stage random sampling	Population based cross-sectional survey	NCEP ATP III, Modified NCEP ATP III and IDF	25.60 (NCEP ATP III), 36.68 (Modified NCEP ATP III), 19.80 (IDF)	Obesity Waist: 11.60 (NCEP ATP III), 31.01 (Modified NCEP ATP III and IDF); High triglyceride: 26.91; Low HDL cholesterol: 85.47; High fasting blood glucose: 30.42 (NCEP ATP III), 44.76 (Modified NCEP ATP III and IDF); Hypertension: 29.12
Jesmin et al. [22]	2012	≥ 15 years	Female	1485; Local Community-Based	Rural	Stratified multi-stage random sampling	Community based cross-sectional study	Modified NCEP ATP III	31.25	Obesity Waist: 31.31; High triglyceride: 26.87; Low HDL cholesterol: 85.05; High fasting blood glucose: 30.57; Hypertension: 29.43
Khanam et al. [21]	2011	≥ 60 years	Both	456; Local Community-Based	Rural	Randomly selected participants from purposively selected blocks	Cross-sectional study	NCEP ATP III	19.5 (male 18.0, female 20.8)	High BMI: 5.3; High triglyceride: 19.50; Low HDL cholesterol: 98.20; High random blood glucose: 13.20; High blood pressure: 49.80
Saquib et al. [17]	2013	≥ 30 years	Both	357; Local	Urban	Multi-stage random sampling	Cross-sectional study	IDF	45.0 (male 29.0, female 61.0)	Prevalence of individual components of MS are presented graphically for male and female. Combined prevalence and numerical values are not presented
Mainuddin et al. [13]	2013	30–60 years	Both	229; Local Hospital-Based	Urban	Random selection	Cross-sectional study	Modified NCEP ATP III, IDF and WHO	72.1 (male 66.0, female 76.7) (Modified NCEP ATP III); 38.9 (male 36.0, female 41.1) (WHO); and 68.6 (male 50.0, female 82.9) (IDF)	NR

The studies selected in this systematic review comprised 14,780 subjects, 80.31% of whom were women and 19.69% men. The minimum age of the participants was 15 years and above in 5 studies, 20 years and above in 2 studies, 30 years and above in 2 studies and 60 years and above in 1 study. Prevalence of metabolic syndrome is reported by all the studies ranged from 8.6% to 72.1%. One study [19] did not report the prevalence and only described the association between parity or gravidity and the prevalence of metabolic syndrome. All the studies where study participants was both male and female, reported prevalence data not only for all but also for males and females separately. One study [18] reported prevalence of metabolic syndrome by age at menarche. Only one study [23] reported age adjusted prevalence of metabolic syndrome, other nine studies reported overall prevalence, gender specific prevalence as well as prevalence in different age groups occasionally. The prevalence of disease is often strongly age-dependent and age adjustment is used to eliminate the effects of age. The pooling of overall prevalence with age adjusted prevalence through meta-analysis could be misleading. However, meta-regression did not identify any significant difference (*p* = 0.656) in overall and age adjusted prevalence of metabolic syndrome, leads us to pool all the prevalences in our meta-analysis.

**Fig. 2:**
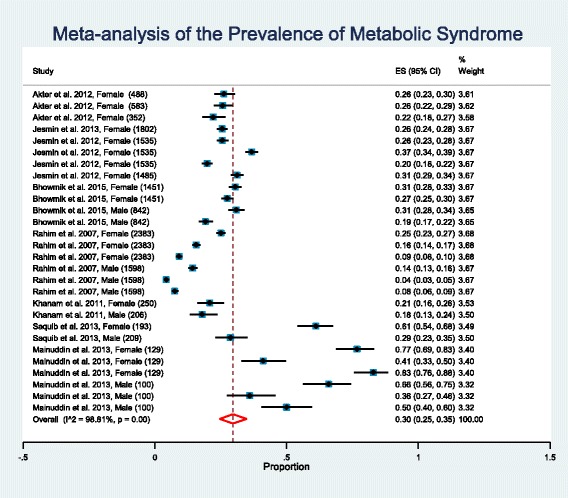
Forest plot of prevalence, with 95% confidence intervals (CIs) of metabolic syndrome in Bangladeshi population. Sample sizes for the studies are given in parentheses

The weighted pooled prevalence of metabolic syndrome regardless of gender, age, and criteria used to define metabolic syndrome, was 30.0% [95% CI: 25% - 35%]. There was a large amount of heterogeneity in the prevalence of metabolic syndrome (I^2^ = 98.81%; Cochran Q-statistic *p* < 0.001; Fig. 2). Meta regression did not identify gender (male vs female, *p* = 0.434) as a potential source of heterogeneity in the overall prevalence of metabolic syndrome but did identify area (urban vs rural, *p* < 0.001) as a source of heterogeneity.

### Subgroup analysis based on the gender of study participants

**Fig. 3:**
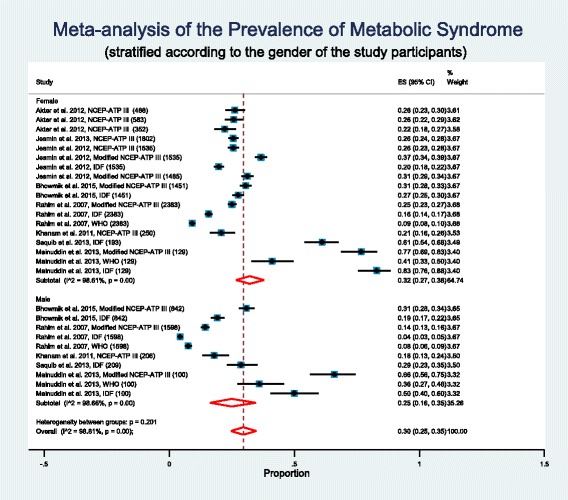
Forest plot of prevalence, with 95% confidence intervals (CIs) of metabolic syndrome in Bangladeshi population, stratified according to the gender of study participants. Sample sizes for the studies are given in parentheses

Almost all studies reported a higher prevalence of metabolic syndrome in females. So we performed a subgroup analysis based on the gender of the study participants (Fig. 3). Weighted pooled prevalence of metabolic syndrome is higher in females, 32% [95% CI: 27% - 38%] compared to males, 25% [95% CI: 16% - 35%]. However meta-regression did not identify this higher prevalence of metabolic syndrome in females statistically significant (p = 0.434). There was significant between study heterogeneity (I^2^ = 98.61%; Cochran Q-statistic *p* < 0.001; Fig. 3) in the prevalence of metabolic syndrome when study participants was female. Meta-regression shows area (urban vs rural, p < 0.001) is the source of heterogeneity. There was also significant between study heterogeneity (I^2^ = 98.66%; Cochran Q-statistic p < 0.001; Fig. 3) in the prevalence of metabolic syndrome when study participants was male. Meta-regression again shows area (urban vs rural, *p* = 0.007) is the source of heterogeneity.

### Subgroup analysis based on criteria used to define metabolic syndrome

**Fig. 4:**
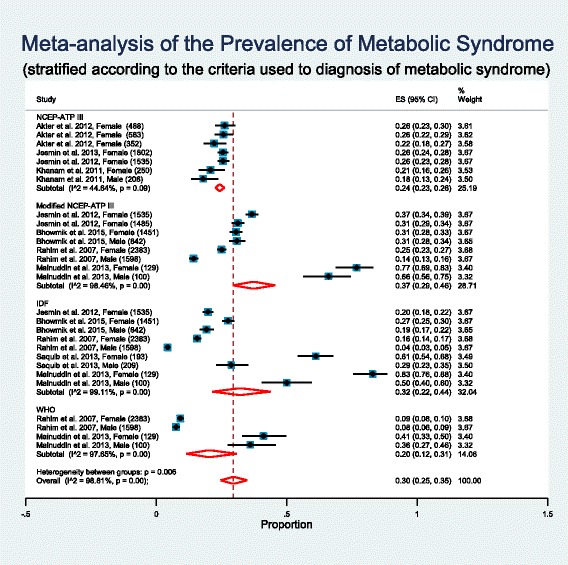
Forest plot of prevalence, with 95% confidence intervals (CIs) of metabolic syndrome in Bangladeshi population, stratified according to the criteria used to diagnosis of metabolic syndrome. Sample sizes for the studies are given in parentheses

There are a number of alternative definitions available for metabolic syndrome and these definitions differed in the proposed components as well as in the cut-off points used for each component, leading to substantial confusion. Recognizing that such differences in metabolic syndrome definition will have an impact on its prevalence, will increase heterogeneity and will raise concern in meaningful pooling of metabolic syndrome prevalence, a subgroup analysis was performed by dividing the studies based on the criteria they used to diagnose metabolic syndrome (Fig. 4). The subgroup analysis will help to explore heterogeneity in the metabolic syndrome prevalence further.

WHO definition emphasized insulin resistance as the major underlying risk factor plus presence of two additional risk factors from obesity, hypertension, high triglycerides, reduced HDL-C level, or microalbuminuria for diagnosis of metabolic syndrome. The NCEP ATP III criteria required the presence of any three of the following five factors as the basis for establishing the diagnosis of metabolic syndrome: abdominal obesity, elevated triglycerides, reduced HDL-C, elevated blood pressure, and elevated fasting glucose. The IDF definition makes the presence of abdominal obesity necessary for diagnosis of metabolic syndrome plus any two additional factors listed in the NCEP ATP III definition. The Modified NCEP ATP III definition maintains the original NCEP ATP III criteria except for minor modifications.

Abdominal obesity measured by waist circumference (WC) is a major component of any metabolic syndrome definition. Cut-off point for WC differs among the metabolic syndrome definitions. The IDF definition uses ethnic-specific WC cut-off points and recommends cut-off levels of ≥90 cm in men and ≥80 cm in women for South Asians. Similar to the IDF definition, Modified NCEP ATP III definition suggested the cut-off levels of ≥90 cm in men and ≥80 cm in women for Asians. In this present review, six studies (those with the Modified NCEP ATP III and IDF definitions) used ethnic-specific WC cut-off of ≥80 cm in women or ≥90 cm in men for South Asians as there are no national cut-off values specific for Bangladesh. Remaining four studies (those with the NCEP ATP III definitions) used WC cut-off of ≥88 cm in women or ≥102 cm in men.

Among the studies that used NCEP ATP III criteria to diagnose metabolic syndrome, weighted pooled prevalence of metabolic syndrome was 24% [95% CI: 23% - 26%] with non-significant little heterogeneity between studies (I^2^ = 44.64%; Cochran Q-statistic *p* = 0.09; Fig. 4). The studies that used Modified NCEP ATP III criteria, weighted pooled prevalence of metabolic syndrome was 37% [95% CI: 29% - 46%] with high heterogeneity between studies (I^2^ = 98.46%; Cochran Q-statistic *p* < 0.001; Fig. 4). In meta-regression gender (male vs female, *p* = 0.854) was not identified as a source of heterogeneity between the studies in this sub group, but area (urban vs rural, *p* = 0.001) was identified as a source of heterogeneity. The studies that used IDF criteria, weighted pooled prevalence of metabolic syndrome was 32% [95% CI: 22% - 44%] with high heterogeneity between studies (I^2^ = 99.11%; Cochran Q-statistic *p* < 0.001; Fig. 4). Area (urban vs rural, *p* = 0.009) was identified as a source of heterogeneity between the studies in this sub group but gender (male vs female, *p* = 0.385) was not identified as a source of heterogeneity. Only two studies used WHO criteria to diagnose metabolic syndrome and the weighted pooled prevalence of metabolic syndrome was 20% [95% CI: 12% - 31%] with high heterogeneity between studies (I^2^ = 97.66%; Cochran Q-statistic *p* < 0.001; Fig. 4). Area (urban vs rural, *p* = 0.012) was identified as a source of heterogeneity between the studies in this sub group but gender (male vs female, *p* = 0.889) was not identified as a source of heterogeneity. An obvious consequence of difference in cut-off points in WC is reflected in weighted pooled prevalence of metabolic syndrome. A much higher pooled prevalence (37% Modified NCEP ATP III, 32% IDF) was observed when WC cut-off of ≥90 cm in men and ≥80 cm in women was used compared to other cut-off point (24% NCEP ATP III). The prevalence of metabolic syndrome varies considerably according to the criteria used to diagnosis of metabolic syndrome. So a subgroup analysis for the prevalence of metabolic syndrome according to the criteria used to diagnosis of metabolic syndrome is probably a better option rather than pooling all the prevalence of metabolic syndrome from different studies.

### Subgroup analysis based on the age of the study participants and assessment of temporal change in metabolic syndrome prevalence

**Fig. 5:**
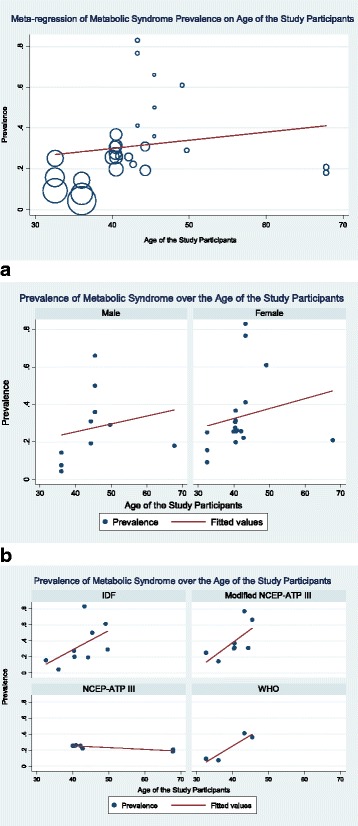
Meta-regression of metabolic syndrome prevalence in Bangladesh on age of the study participants. **a** Prevalence of metabolic syndrome in Bangladesh over the age of the study participants (stratified by the gender of the study participants). **b** Prevalence of metabolic syndrome in Bangladesh over the age of the study participants (stratified by the definition of metabolic syndrome)

The prevalence of disease is often strongly age-dependent, so an age effect on the prevalence of the metabolic syndrome is evident and need to be explored. Beside the overall prevalence, most of the studies (8 out of 10) provided age stratified prevalence of metabolic syndrome. However the age grouping in different studies were different which restricts us providing weighted pooled prevalence of metabolic syndrome in Bangladesh among the different age groups. Instead, we divide the studies based on the mean age of the study participants and performed a meta-regression. Studies that did not report the mean age of the study participants, grouped data mean calculation formula is used to calculate the mean. For open end age intervals (e.g. < 25 or 50 +) where mean is not possible to calculate, we used median and calculated median age of the study participants. Meta-regression shows, a significant (*p* = 0.006, mean age cut-off ≤40 years) higher weighted pooled prevalence (36% versus 14%) of metabolic syndrome in study participants with mean age greater than 40 years. Overall prevalence of metabolic syndrome increases 0.4% (*p* = 0.38; Fig. 5) for every one year increase in mean age of the study participants. Such increase in metabolic syndrome prevalence over the age of the participants is quite similar in male and female (Fig. 5a), however, little different when definition of metabolic syndrome varies (Fig. 5b). A decrease in metabolic syndrome prevalence over the age of the study participants was observed for NCEP ATP III definition (Fig. 5b).

**Fig. 6:**
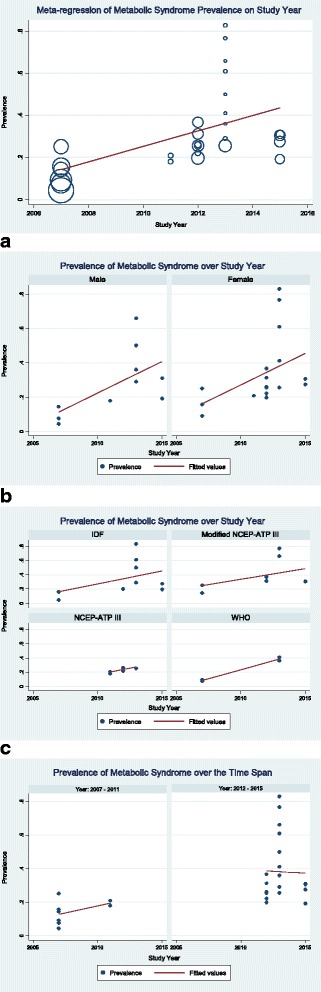
Meta-regression of metabolic syndrome prevalence in Bangladesh on the study year. **a** Prevalence of metabolic syndrome in Bangladesh over the study year (stratified by the gender of the study participants).**b** Prevalence of metabolic syndrome in Bangladesh over the study year (stratified by the definition of metabolic syndrome). **c** Prevalence of metabolic syndrome in Bangladesh over the different time span

As time of the study can affect the prevalence of metabolic syndrome, we explored such temporal change in metabolic syndrome prevalence. Studies included in present review has a time span of eight years from 2007 to 2015. We split the studies into two groups (studies that conducted before the year 2012 and the studies that conducted in the year 2012 and afterwards). Meta-regression shows, a significant (*p* = 0.002) higher weighted pooled prevalence (37% versus 14%) of metabolic syndrome in studies conducted in the year 2012 and afterwards. Overall prevalence of metabolic syndrome increases 3.68% (*p* = 0.006; Fig. 6) for every one year increase in time of the study conducted. Such increase in metabolic syndrome prevalence over the time of the study conducted is quite similar in male and female (Fig. 6a) and also when definition of metabolic syndrome varies (Fig. 6b). An increasing trend in pooled prevalence of metabolic syndrome is observed during 2007–2011 time period while a slightly decreasing trend is observed during 2012–2015 time period (Fig. 6c).

### Prevalence of individual components of metabolic syndrome

Prevalence of individual components of metabolic syndrome is reported in 6 studies, of which one study [17] presented prevalence of individual components of metabolic syndrome graphically for males and females but did not present its numerical values. One study [23] reported only the prevalence of elevated waist circumference, hypertension, and mean fasting plasma glucose. Instead of reporting prevalence of low HDL cholesterol and high triglyceride, it reported prevalence of dyslipidemia. Four studies did not report the prevalence of individual components. Five studies reported the prevalence of abdominal obesity. In these five studies, the weighted pooled prevalence of abdominal obesity was 18.0% [95% CI: 11.0% - 27.0%]. Though a markedly different pooled prevalence of abdominal obesity (8% versus 27%) was observed when WC cut-off values shifted from ≥88 cm in women or ≥102 cm in men to ≥80 cm in women or ≥90 cm in men.

The prevalence of hypertension was mentioned in five studies and the weighted pooled prevalence of hypertension was found 30% [95% CI: 21% - 39%]. The weighted pooled prevalence of high fasting glucose was 28% [95% CI: 15% - 42%] based on five studies. Low HDL cholesterol was reported in four studies and weighted pooled prevalence was observed 89% [95% CI: 83% - 94%]. Four studies reported the prevalence of high triglyceride and the weighted pooled prevalence was 26% [95% CI: 23% - 29%].

### Study quality assessment

Quality of the studies was assessed according to the set of criteria based on Joanna Briggs Institute guidance on conducting prevalence and incidence reviews [15, 16] and are summarized in Table 3. A set of eight criteria was used to assess the quality of the studies. The sample was representative of the target population in all studies aside from one investigation [13]. Study participants were recruited in an appropriate way in all the studies. The sample size was adequate only in 3 (30%) studies. Study subjects and setting was described in detail in 6 (60%) studies. The data analysis was conducted with sufficient coverage of the identified sample in 6 (60%) studies. An objective, standard criterion was used for reliably measure the condition in almost all studies with one exception [22]. All the studies accounted for important confounding factors and subgroups.

**Table 3:** Study quality assessment of studies that evaluated the prevalence of metabolic syndrome (MS) in the Bangladeshi population

Study	Was the sample representative of the target population?	Were study participants recruited in an appropriate way?	Was the sample size adequate?	Were the study subjects & setting described in detail?	Was the data analysis conducted with sufficient coverage of the identified sample?	Were objective, standard criteria used for measurement of the condition?	Was the condition measured reliably?	Are all the important confounding factors/ subgroups/ differences identified and accounted for?
Akter et al. (2012) [18]	Yes	Yes	Yes	Yes	Yes	Yes	Yes	Yes
Akter et al. (2013) [19]	Yes	Yes	Not Clear	Yes	Not Clear	Yes	Yes	Yes
Bhowmik et al. (2015) [23]	Yes	Yes	Not Clear	Yes	Not Clear	Yes	Yes	Yes
Rahim et at. (2007) [14]	Yes	Yes	Not Clear	No	Not Clear	Yes	Yes	Yes
Jesmin et al. (2013) [20]	Yes	Yes	Yes	Yes	Yes	Yes	Yes	Yes
Jesmin et al. (2012) [24]	Yes	Yes	Yes	Yes	Yes	Yes	Yes	Yes
Jesmin et al. (2012) [22]	Yes	Yes	Not Clear	No	Yes	Not Clear	Not Clear	Yes
Khanam et al. (2011) [21]	Yes	Yes	Not Clear	No	Not Clear	Yes	Yes	Yes
Saquib et al. (2013) [17]	Yes	Yes	Not Clear	Yes	Yes	Yes	Yes	Yes
Mainuddin et al. (2013) [13]	No	Yes	Not Clear	No	Yes	Yes	Yes	Yes

### Publication bias

**Fig. 7:**
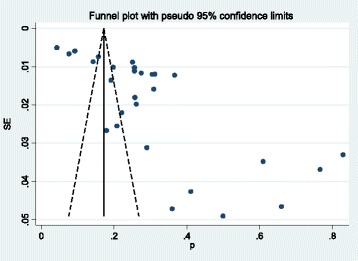
Funnel plot for the publication bias of the studies that evaluated the prevalence of metabolic syndrome in ; Bangladeshi population

The funnel plot indicates the existence of asymmetry and publication bias (Fig. 7) and Egger test (*p* < 0.001) suggested the presence of small study effects, in which studies of smaller cohorts reporting higher prevalence of metabolic syndrome.

## Discussion

The present systematic review provides summary estimates for the prevalence of metabolic syndrome in the Bangladeshi population. The findings of this review suggests that, the weighted pooled prevalence of metabolic syndrome are between 20.0 and 37.0% depending on the criteria used to define metabolic syndrome. Prevalence was highest (37%) when Modified NCEP ATP III criteria was used, while it was lowest (20%) when WHO criteria was used. The observed overall pooled prevalence of metabolic syndrome (30%) in Bangladesh was slightly higher than the prevalence estimated around the world (between 20% and 25%) [1].

While comparing our findings with reports from other regions, we see mixed results. A systematic review of Latin American countries showed a prevalence of 24.9% (ATP III)] [25]. A combined prospective cohort studies of Europe reported the overall prevalence of metabolic syndrome 15% (WHO) in nondiabetic adults [26]. The prevalence of metabolic syndrome among adults according to a national survey of US is 35% (NCEP ATP III) and 39% (IDF) [27]. In a systematic review conducted on gulf countries, the reported prevalence of metabolic syndrome with ATP III definition ranged from 19.5–37.2% (men) and from 13.5–42.7% (women), while with the IDF definition, the prevalence ranged from 18.4–36.2% (men) and from 16.1–45.9% (women) [28]. The prevalence in a sub-Saharan African setting varies from 0 to 7.3% according to different definition of metabolic syndrome [29]. A systematic review on prevalence of metabolic syndrome in Asia-pacific region reported a prevalence of 11.9% (NCEP ATP III) to 49.0% (modified NCEP ATP III) [30]. The weighted mean prevalence of metabolic syndrome was 14.0% (WHO), 26.1% (ATPIII), 29.8% (IDF) and 32.5% (modified ATPIII) in South Asia according to a systematic review [31].

This study demonstrated low HDL cholesterol as the most frequent individual component of metabolic syndrome with weighted pooled prevalence of 89%. High blood pressure was shown the second most prominent metabolic syndrome component with weighted pooled prevalence of 30%. High fasting glucose was the third most prevalent metabolic syndrome component in our study (28%). The underlying factors behind the increased prevalence of low HDL, high blood pressure, and high fasting glucose among Bangladeshi population could be multifarious. However, regional changes in disease patterns from communicable to non-communicable diseases, an increasing trend of urbanization and fascination for adopting western lifestyle could influence such high prevalence [32, 33]. Rapid and unplanned urbanization is responsible for lifestyle change such as physical inactivity, changes in diet and stress and is closely linked with higher prevalence of metabolic syndrome and is evidenced by the higher weighted pooled prevalence of metabolic syndrome observed among urban population (56% compared to 21% in rural population).

We observed high between-study-heterogeneity across the studies. One possible source of heterogeneity is the area (urban/rural) where study was conducted. Prevalence of metabolic syndrome also differed in males and females and it is more prevalent in females as identified by subgroup analysis. Within the male and female subgroups, there was high between-study-heterogeneity on prevalence of metabolic syndrome. As different studies used different criteria to diagnosis of metabolic syndrome, a subgroup analysis based on the criteria used to define metabolic syndrome was performed to assess the heterogeneity on the prevalence of metabolic syndrome among the studies. However, heterogeneity was still observed within the subgroups, and study area and gender was identified as the source of heterogeneity within the subgroups.

Evidence of small study effect was detected (*p* < 0.001), in which smaller studies reported higher prevalence of metabolic syndrome. Publication bias was also evident from the asymmetry on the funnel plot. Study quality assessment shows most of the studies fail to meet the criteria “adequate sample size” for their studies. Also in some studies the data analysis was conducted without sufficient coverage of the identified sample, which is a concern.

The strength of this study is the comprehensiveness of the process, which included a search of three different databases, well-defined inclusion/exclusion criteria, and extensive use of reference lists. Thus it is unlikely that any relevant studies would have been missed. Nonetheless, there are limitations to our systematic review and meta-analysis. We could not consider non- English publications as well as very local level journals that are not available through the major academic databases. Also a point needs to be noted that there is no uniformity of metabolic syndrome definitions, age groups, waist circumference cut-offs, and study settings in the studies included in the present review, resulting in limitations in comparability.

## Conclusion

To our knowledge, this is the first comprehensive report to systematically evaluate the scientific literature on the prevalence of metabolic syndrome in Bangladesh. Despite differences in diagnostic criteria, gender, age and geographic area of subjects studied, the prevalence of metabolic syndrome is high and rising in Bangladesh according to this systematic review and recommends an urgent attention from both the clinical and public health viewpoint. Hence strategies aimed at primary prevention are required to mitigate a further increase in the prevalence and for the reduction of the morbidity and mortality associated with metabolic syndrome. It is also extremely important to explore possible risk factors, especially those related to lifestyle, which need to be addressed. Knowledge of these factors may be informative in the monitoring of metabolic syndrome and could contribute to planning and prevention strategies to tackle this problem.

## Additional file

Additional file 1: **Figure S1.** The regions where studies conducted to identify metabolic syndrome prevalence in Bangladesh. (DOCX 83 kb)

## Abbreviations

BMI: Body mass index
HD: High density lipoprotein
IDF: International Diabetes Federation
MS: Metabolic syndrome
NCEP-ATP III: National Cholesterol Education Program Adult Treatment Panel III
WC: Waist circumference
WHO: World Health Organization
