# Supplementary material for: Prevalence of metabolic syndrome in Bangladesh: a systematic review and meta-analysis of the studies
Source: BMC Public Health. 2018 Mar 2;18:308. doi: 10.1186/s12889-018-5209-z (PMC5833131; doi:10.1186/s12889-018-5209-z)
Supplement: Supplementary file 1 — Figure S1. The regions where studies conducted to identify metabolic syndrome prevalence in Bangladesh. (DOCX 83 kb) [file 12889_2018_5209_MOESM1_ESM.docx]

**Figure S1.**


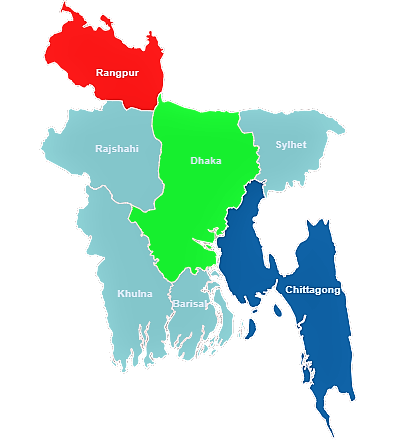


**Figure S1.** The regions where studies conducted to identify metabolic syndrome prevalence in Bangladesh.

No studies conducted =

111

Five studies conducted (weighted pooled prevalence, 27%) =

Four studies conducted (weighted pooled prevalence, 32%) =

One study conducted (weighted pooled prevalence, 19%) =

1111
